# Role of provocation and exercise imaging for the identification of candidates for cardiac myosin inhibitors

**DOI:** 10.1093/eschf/xvag087

**Published:** 2026-03-25

**Authors:** Jonas Erzeel, Sebastiaan Dhont, Marnicq van Es, Duhan Ulgar, Philippe Bertrand, Wilfried Mullens, Pieter Martens

**Affiliations:** Department of Cardiology, Ziekenhuis Oost-Limburg, Genk, Belgium; Faculty of Medicine and Life Sciences/LCRC, Hasselt University, Diepenbeek, Belgium; Department of Cardiology, Ziekenhuis Oost-Limburg, Genk, Belgium; Faculty of Medicine and Life Sciences/LCRC, Hasselt University, Diepenbeek, Belgium; Department of Cardiology, Ziekenhuis Oost-Limburg, Genk, Belgium; Faculty of Medicine and Life Sciences/LCRC, Hasselt University, Diepenbeek, Belgium; Department of Cardiology, Ziekenhuis Oost-Limburg, Genk, Belgium; Department of Cardiology, Ziekenhuis Oost-Limburg, Genk, Belgium; Faculty of Medicine and Life Sciences/LCRC, Hasselt University, Diepenbeek, Belgium; Department of Cardiology, Ziekenhuis Oost-Limburg, Genk, Belgium; Faculty of Medicine and Life Sciences/LCRC, Hasselt University, Diepenbeek, Belgium; Department of Cardiology, Ziekenhuis Oost-Limburg, Genk, Belgium; Faculty of Medicine and Life Sciences/LCRC, Hasselt University, Diepenbeek, Belgium

**Keywords:** Hypertrophic cardiomyopathy, Left-ventricular outflow tract obstruction, Exercise echocardiography, Cardiac myosin inhibitors, Exercise-induced obstruction

## Abstract

**Aims:**

Left ventricular outflow tract obstruction (LVOTO) drives symptoms and functional limitation in obstructive hypertrophic cardiomyopathy (oHCM). Some patients may only show treatment-qualifying obstruction during exercise echocardiography, yet their clinical profile and response to cardiac myosin inhibition are not well defined. This study compared the characteristics and therapeutic response of patients requiring exercise echocardiography to establish eligibility for mavacamten versus those meeting criteria at rest or during Valsalva.

**Methods and results:**

A single-centre retrospective cohort of 56 symptomatic oHCM patients treated with mavacamten was evaluated. LVOTO was assessed at rest, with Valsalva, and during exercise; patients were classified as ‘exercise’ or ‘non-exercise’ LVOTO based on the provocation manoeuvre eliciting a qualifying gradient (≥50 mmHg). Haemodynamic (Valsalva LVOT gradient) and symptomatic (NYHA class) response were assessed at 12 and 24 weeks. A total of 42.9% qualified for mavacamten exclusively during exercise echocardiography. Although resting and Valsalva gradients were lower by definition, these patients showed similar baseline functional limitation and exercise capacity (pVO2; 17.9 ± 7.4 vs. 16.8 ± 5.5 mL/kg/min, *P* = .550). By 24 weeks, most patients in both groups achieved non-obstructive gradients (<30 mmHg; 93.3% vs. 96.4%, *P* = .646) and NYHA class improvement (75.0% vs. 93.3%, *P* = .266), without significant between-group differences.

**Conclusion:**

Patients requiring exercise echocardiography to document treatment-qualifying LVOTO do not exhibit a milder disease phenotype and derive similar treatment benefits from mavacamten compared to those with resting or Valsalva-provoked obstruction. Exercise echocardiography identifies a substantial proportion of symptomatic HCM patients with significant LVOTO missed by resting assessment and is essential for guiding treatment eligibility.

## Graphical Abstract

**Graphical Abstract:**
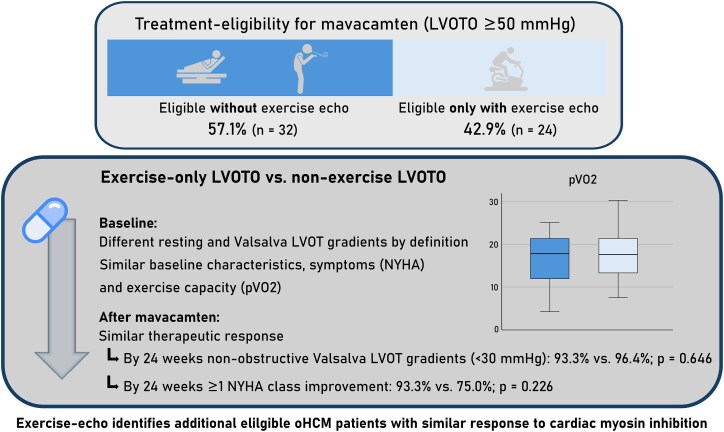

## Introduction

Hypertrophic cardiomyopathy (HCM) is a common inherited cardiac disorder characterized by left ventricular hypertrophy and a heterogeneous clinical presentation.^1,2^ A substantial proportion of patients develop left ventricular outflow tract obstruction (LVOTO), which is a major determinant of symptoms, exercise intolerance, and adverse outcomes.^3–6^ Obstructive physiology in HCM may be present at rest or may occur only under dynamic conditions such as physiological provocation or exercise, underscoring the importance of comprehensive echocardiographic assessment for accurate disease characterization and detection of clinically relevant obstruction.^3,5–7^

Mavacamten, a selective cardiac myosin inhibitor, has emerged as an effective disease-specific therapy for symptomatic obstructive hypertrophic cardiomyopathy (oHCM). Randomized trials have demonstrated that mavacamten reduces left ventricular outflow tract (LVOT) gradients, improves functional capacity, alleviates symptoms, and reduces the need for septal reduction therapy in patients with resting or provokable obstruction.^8–10^ Consequently, current treatment algorithms and reimbursement criteria require documentation of significant LVOTO, typically defined by a peak gradient ≥50 mmHg at rest or with provocation.^1,2^ In clinical practice however, a subset of patients may only meet this LVOT threshold during exercise echocardiography, raising questions regarding their clinical phenotype and expected treatment response. Specifically, it remains uncertain whether patients with obstruction provoked exclusively by exercise represent a milder disease phenotype or if they derive similar benefit from cardiac myosin inhibition. Real-world data addressing this population are limited, most available evidence is derived from clinical trials with strict inclusion criteria and limited representation of patients with isolated exercise-induced obstruction. Consequently, the haemodynamic and symptomatic response to mavacamten in this specific subgroup has not been well characterized.

This retrospective, single-centre study evaluated consecutive patients with oHCM initiated on mavacamten. We sought to determine (1) the frequency with which exercise echocardiography was required to establish eligibility for mavacamten, (2) whether patients with isolated exercise-induced LVOTO exhibit a milder clinical or functional phenotype at baseline, and (3) whether the magnitude of haemodynamic and symptomatic response to mavacamten differs between patients with resting versus exercise-induced LVOTO at baseline.

## Methods

### Study design and population

This retrospective, single-centre study included all consecutive adult patients with oHCM who initiated treatment with macavamtem at Ziekenhuis Oost-Limburg (Genk, Belgium) between January 2024 and November 2025. Diagnosis of HCM was established by the treating cardiologist based on the presence of otherwise unexplained left ventricular hypertrophy, defined as a wall thickness ≥15 mm (or ≥13 mm in the presence of familial HCM) in at least one myocardial segment.^1,2^ Treatment eligibility for mavacamten adhered to guideline treatment algorithms and the Belgian reimbursement criteria (Supplemental Text S1 &; S2). In brief, patients were eligible for mavacamten if they had obstructive HCM defined as a peak LVOT gradient ≥50 mmHg (either at rest, after Valsalva or during exercise), a left ventricular ejection fraction (LVEF) ≥55%, and persistent symptoms despite guideline-directed medical therapy (New York Heart Association [NYHA] functional class II-III).^2,11^ Institutional approval was obtained from the local ethics committee, and due to the retrospective design and use of anonymized data, the requirement for written informed consent was waived.

### Echocardiographic assessment

Comprehensive transthoracic echocardiography was performed in all patients to detect and quantify obstructive physiology at baseline. Peak LVOT gradients were measured using continuous-wave Doppler aligned with the direction of systolic flow, and values were reported as peak instantaneous gradients. Obstruction was assessed under three conditions: at rest, during provocation with the Valsalva manoeuvre, and during exercise echocardiography when clinically indicated (including all patients with resting or Valsalva-provoked LVOTO <50 mmHg, as well as selected patients with higher gradients). Resting studies were obtained on left lateral decubitus position. For Valsalva provocation, the peak gradient achieved at maximal strain was recorded. Exercise echocardiography was performed using a semi-supine bicycle protocol described previously,^12,13^ with LVOT gradients measured at peak exercise or in early recovery. Patients were subsequently grouped according to the condition in which the qualifying LVOT gradient of ≥50 mmHg was first documented. Those meeting the threshold at rest were classified as the resting LVOTO group, those who did not qualify at rest but exceeded the threshold during the Valsalva manoeuvre were assigned to the Valsalva LVOTO group, and those requiring exercise echocardiography were assigned to the exercise LVOTO group. For subsequent analyses, the rest and Valsalva groups were merged into a single ‘non-exercise LVOTO’ group, which was compared with the exercise LVOTO group to evaluate baseline differences and treatment response. During follow-up, peak LVOT gradients were reassessed at rest and during the Valsalva manoeuvre, with particular focus on the latter on this study.

### Mavacamten treatment and follow-up

All patients initiated mavacamten at a starting dose of 2.5 mg once daily, in accordance with the European product label and Belgian reimbursement criteria (in the setting of unknown CYP2C19 status). Dose titration was performed at prespecified intervals of 4, 8, and 12 weeks, and subsequently every 1 to 3 months, based on LVEF and peak LVOT gradient, with adjustments made at the discretion of the treating cardiologist in accordance to the Summary of Product Characteristics. Titration was permitted to dosage levels of 2.5 mg, 5 mg, 10 mg, and a maximum dose of 15 mg once daily. Treatment interruption or dose reduction was performed when LVEF fell below guideline-recommended thresholds or when clinically indicated. Clinical assessment was performed at baseline and during follow-up visits, with symptoms evaluated using patient-reported NYHA functional classification. Changes in NYHA class were extracted from the medical chart.

### Statistical analysis

Continuous variables are presented as mean ± standard deviation and were compared between groups using independent t-tests or Mann-Whitney *U* tests, as appropriate. Categorical variables are reported as counts and percentages and were compared using the Chi-square test or the Fisher’s exact test, as appropriate. Valsalva-provoked LVOT gradients at 12 and 24 weeks after mavacamten initiation were analysed both as categorical and continuous variables. For categorical analysis, gradients were dichotomized using a threshold of <30 mmHg—consistent with the definition of non-obstructive physiology in standard clinical practice—and compared between groups using the Chi-square test or Fisher’s exact test, as appropriate. Continuous LVOT gradients at 12 and 24 weeks were compared between groups using analysis of covariance (ANCOVA) adjusted for baseline values and reported as adjusted means with standard errors. Symptomatic response was evaluated by assessing the proportion of patients with an improvement of at least one NYHA functional class at 12 and 24 weeks, with between-group differences compared using the Chi-square test. Longitudinal evolution of NYHA class was examined using a generalized estimating equations (GEE) model. Distributions of NYHA class at each follow-up timepoint were compared between groups using the Chi-square test or Fisher’s exact test, as appropriate The achieved mavacamten dose at 12 and 24 weeks was compared between groups using the Chi-square test or Fisher’s exact test, as appropriate. A two-sided *P*-value <.05 was considered statistically significant. All statistical analyses were performed using SPSS version 30.0 (IBM Corp., Armonk, N.Y., USA).

## Results

### Baseline characteristics and qualifying LVOT obstruction

**Figure 1:**
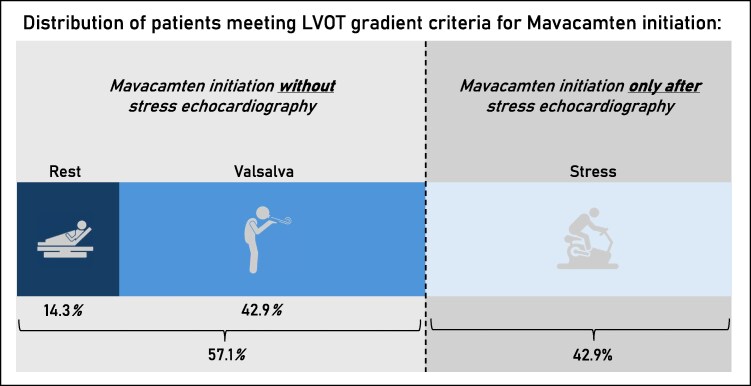
Distribution of echocardiographic conditions prompting mavacamten initiation. Proportion of patients meeting treatment-qualifying left ventricular outflow tract obstruction (LVOTO) criteria based on resting echocardiography, Valsalva-provocation, or exercise echocardiography. In 57.1% of patients, treatment eligibility was established without the need for exercise echocardiography (rest: 14.3%; Valsalva: 42.9%), whereas in 42.9% of patients, a qualifying LVOT gradient was documented exclusively during exercise echocardiography

A total of 56 consecutive patients with oHCM initiated mavacamten and were included in the analysis. Of these, 8 (14.3%) met treatment eligibility criteria on resting LVOT gradients, 24 (42.9%) based on Valsalva-provoked gradients, and 24 (42.9%) exclusively during exercise echocardiography (*Figure 1*). For comparative analyses, patients were grouped into those who required exercise echocardiography to demonstrate eligibility (exercise LVOTO group, *n* = 24 [42.9%]) and those who met criteria with resting or Valsalva measurements (non-exercise LVOTO group, *n* = 32 [57.1%]).

Baseline characteristics are summarized in *Table 1*. As expected by design, in patients requiring exercise echocardiography for eligibility, baseline LVOT gradients at rest and during the Valsalva manoeuvre were substantially lower compared with those in the non-exercise group (16.3 ± 10.1 vs. 39.2 ± 19.5 and 34.8 ± 11.4 vs. 78.6 ± 28.2, respectively; *P* < .001 for both), confirming that these patients exhibited treatment-eligible obstruction exclusively during exercise. In contrast, peak LVOT gradients during exercise were similar between groups (87.9 ± 49.5 vs. 85.4 ± 21.6 mmHg; *P* = .314). Demographic, clinical, and structural characteristics were otherwise comparable between the groups. Age, sex distribution, comorbidities, and the prevalence of angina were similar. Family history of HCM and the proportion of patients carrying a pathogenic gene variant did not differ significantly. Baseline NYHA functional class was also similar, with the majority of patients in both groups presenting with NYHA class II symptoms. Echocardiographic markers of structural disease, including LVEF, interventricular septal thickness, and left atrial diameter, were comparable between groups.

**Table 1:** Baseline characteristics according to the echocardiographic condition prompting mavacamten initiation

	Overall (*n* = 56)	Non-exercise LVOTO (*n* = 32)	Exercise LVOTO (*n* = 24)	*P* value
*Demographics*				
Age (years)	64.5 ± 12.9	65.3 ± 13.0	63.4 ± 12.9	.882
Male sex	32 (57.1)	19 (59.4)	13 (54.2)	.697
*Family history & Genetics*				
Family history of HCM	10 (17.9)	8 (25.0)	2 (8.3)	.107
HCM genetic testing performed	42 (75.0)	24 (75.0)	18 (75.0)	1.000
Pathogenic gene variant	11 (26.2)	5 (20.8)	6 (33.3)	.483
*Symptoms & Functionality*				
NYHA functional class				.437
2	40 (71.4)	21 (65.6)	19 (79.2)	
3	15 (26.8)	10 (31.3)	5 (20.8)	
4	1 (1.8)	1 (3.1)	0 (0.0)	
Angina	14 (25.5)	8 (25.0)	6 (26.1)	.927
*Comorbidities*				
Hypertension	34 (60.7)	17 (53.1)	17 (70.8)	.179
Obesity	20 (35.7)	12 (37.5)	8 (33.3)	.747
Diabetes	7 (12.5)	4 (12.5)	3 (12.5)	1.000
Heart Failure	3 (5.4)	1 (3.1)	2 (8.3)	.392
AF	8 (14.3)	4 (12.5)	4 (16.7)	.659
CAD	15 (26.8)	8 (25.0)	7 (29.2)	.728
OSAS	8 (14.5)	4 (12.9)	4 (16.7)	.695
CKD	10 (17.9)	5(15.6)	5 (20.8)	.615
Stroke	2 (3.6)	2 (6.3)	0 (0.0)	.212
*Background HCM treatment*				
Beta-blocker	45 (80.4)	26 (81.3)	19 (79.2)	.846
Non-dihydropyridine CCB	10 (17.9)	5 (15.6)	5 (20.8)	.615
*History of sudden cardiac death and implantable cardiac defibrillator*				
SCD	1 (1.8)	1 (3.1)	0 (0.0)	1.000
ICD	11 (19.6)	8 (25.0)	3 (12.5)	.244
*Echocardiographic parameters*				
LVEF	73.9 ± 14.3	72.3 ± 17.2	76.6 ± 6.8	.411
IVS diameter	18.2 ± 4.5	18.4 ± 3.5	18.0 ± 6.0	.831
LA diameter	41.0 ± 5.5	41.6 ± 5.6	40.1 ± 5.5	.425
LVOTO at rest	29.4 ± 19.7	39.2 ± 19.5	16.3 ± 10.1	**<**.**001**
LVOTO during Valsalva	59.9 ± 31.3	78.6 ± 28.2	34.8 ± 11.4	**<**.**001**
LVOTO during exercise	87.1 ± 41.9	85.4 ± 21.6	87.9 ± 49.5	.314

Values are n (%) or mean ± standard deviation for continuous variables.

AF, atrial fibrillation; CAD, coronary artery disease; CCB, calcium channel blocker; CKD, chronic kidney disease; HCM, hypertrophic cardiomyopathy; ICD, implantable cardiac defibrillator; IVS, intraventricular septum; LA, left atrial; LVEF, left ventricular ejection fraction; LVOTO, left ventricular outflow tract obstruction; NYHA, New York Heart Association; OSAS, obstructive sleep apnoea syndrome; SCD, sudden cardiac death

### Functional capacity and cardiopulmonary exercise testing

**Figure 2:**
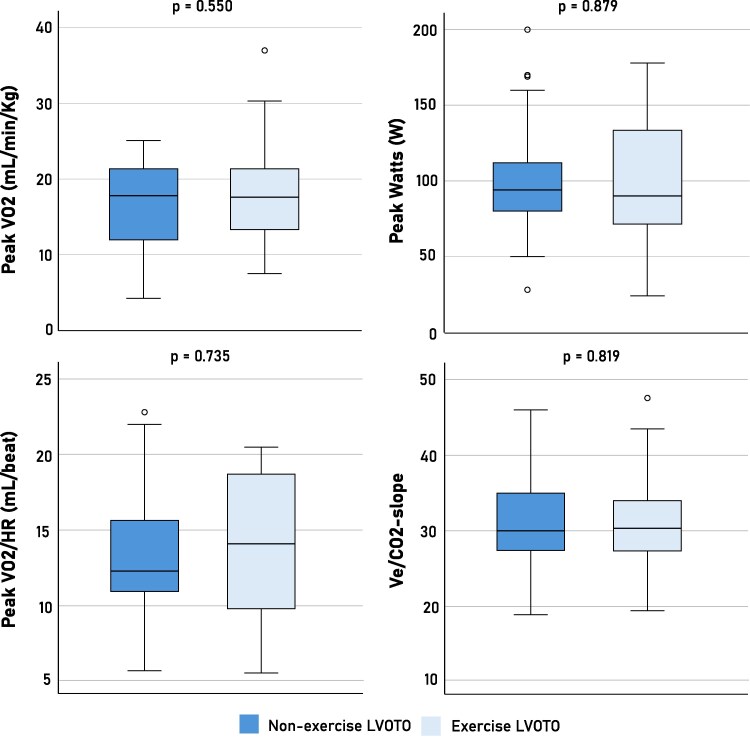
Cardiopulmonary exercise testing characteristics according to echocardiographic condition prompting mavacamten initiation. Box-and-whisker plots compare peak oxygen uptake (VO_2_), peak workload, peak VO_2_/heart rate (VO_2_/HR), and ventilatory efficiency (Ve/VCO_2_ slope) between patients who met treatment eligibility without exercise echocardiography (non-exercise LVOTO) and those who qualified exclusively during exercise echocardiography (exercise LVOTO). No significant differences were observed between groups for any CPET parameter, indicating a comparable degree of functional limitation. *P*-values refer to between-group comparisons

Cardiopulmonary exercise testing (CPET) characteristics are presented in *Table 2*, with corresponding distributions illustrated in *Figure 2*. Baseline CPET data were available in 50 out of 56 patients (89.3%). Measures of exercise capacity were similar between the exercise and non-exercise LVOTO groups and demonstrated the physiological impairment typically observed in symptomatic oHCM. Peak VO_2_ did not differ significantly between groups (17.9 ± 7.4 vs. 16.8 ± 5.5 mL/kg/min; *P* = .550), nor did peak workload (99.0 ± 46.4 vs. 100.9 ± 40.5 W; *P* = .879). When expressed relatively to predicted values, peak VO2 was mildly reduced in both groups averaging 81.3 ± 28.7% in the exercise LVOTO group and 76.7 ± 21.5% in the non-exercise LVOTO group (*P* = .517), indicating comparable degree of aerobic limitation. Ventilatory efficiency was modestly impaired in both groups, with Ve/VCO2-slopes of 31.3 ± 6.4 and 30.9 ± 5.9 for the exercise and non-exercise LVOTO groups, respectively (*P* = .819).

**Table 2:** Cardio-pulmonary exercise testing characteristics according to the echocardiographic condition prompting mavacamten initiation

	Non-exercise LVOTO (*n* = 32)	Exercise LVOTO (*n* = 24)	*P* value
CPET performed	27 ± 84.4	23 ± 95.8	.223
Peak Workload (W)	100.9 ± 40.5	99.0 ± 46.4	.879
Peak VO2 (mL/min/Kg)	16.8 ± 5.5	17.9 ± 7.4	.550
% Predicted	76.7 ± 21.5	81.3 ± 28.7	.517
Heart rate (bpm)	109.4 ± 24.0	114.1 ± 15.6	.411
% Predicted	69.6 ± 13.3	72.1 ± 8.2	.427
Peak VO2/HR (mL/beat)	13.4 ± 4.0	13.8 ± 4.8	.735
VO2/Watt-slope (mL/min/W)	10.5 ± 3.4	11.3 ± 3.5	.396
Ve/VCO2-slope	30.9 ± 5.9	31.3 ± 6.4	.819
RER	1.0 ± 0.1	0.98 ± 0.1	.501

Values are mean ± standard deviation.

Bpm, beats-per-minute; CPET, cardiopulmonary exercise testing; HR, heart rate; RER, respiratory exchange ratio; Ve/VCO_2_ slope, ventilatory equivalent for carbon dioxide slope; VO_2_, peak oxygen uptake

### LVOT gradient response to mavacamten

**Figure 3:**
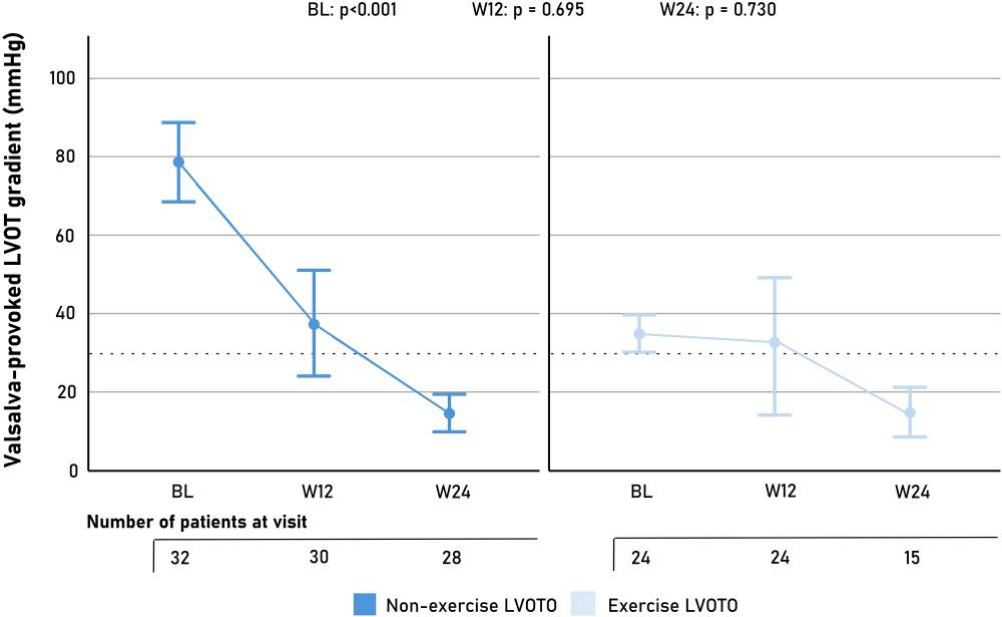
Valsalva-provoked LVOT gradient during mavacamten therapy. Adjusted mean LVOT gradients at baseline (BL), 12 weeks (W12), and 24 weeks (W24) are shown for patients who met treatment eligibility without exercise echocardiography (non-exercise LVOTO) and for those who qualified exclusively during exercise echocardiography (exercise LVOTO). Error bars represent 95% confidence intervals. The dashed horizontal line indicates the conventional threshold for obstructive physiology (30 mmHg). Numbers below the x-axis denote the number of patients with available echocardiographic data at each visit. *P*-values refer to between-group comparisons

Follow-up data at 12 and 24 weeks were available in 54 patients (96.4%) and 43 patients (76.8%), respectively. One patient discontinued treatment due to drug-related adverse effects, two patients did not attend their follow-up visits at our institution, and the remaining patients without follow-up data had initiated treatment less than 12 or 24 weeks before data extraction. By 12 weeks, the proportion of patients achieving a Valsalva-provoked LVOT gradient <30 mmHg did not significantly differ between groups (62.5% in the exercise LVOTO group vs. 46.7% in the non-exercise LVOTO group; *P* = .246; *Table 3*). By 24 weeks, rates of obstruction resolution increased further in both groups (93.3% vs. 96.4%; *P* = .646), with no significant difference between patients with non-exercise versus exercise-induced LVOTO. Cross-sectional analyses adjusted for the baseline gradient showed no significant differences in Valsalva-provoked LVOT gradient between groups at either 12 or 24 weeks (adjusted means 31.9 ± 9.0 vs. 37.3 ± 7.7 and 16.3 ± 3.2 vs. 14.8 ± 2.1; *P* = .695 and .730, respectively; *Figure 3*). Thus, despite differing baseline physiology, the groups exhibited a similar degree of obstruction at both follow-up visits after adjustment for baseline.

**Table 3:** LVOT obstruction and functional outcomes according to the echocardiographic condition prompting mavacamten initiation

		Non-exercise LVOTO	Exercise LVOTO	Difference (95% CI)	*P* value
LVOTO <30 mmHg	Week 12	14/30 (46.7%)	15/24 (62.5%)	15.8% (−10.5%–42.1%)	.246
	Week 24	27/28 (96.4%)	14/15 (93.3%)	−3.1% (−17.4%–11.2%)	.646
Adjusted LVOTO (mmHg)	Week 12	37.3 ± 7.7	31.9 ± 9.0	−5.4 (−32.6–21.9)	.695
	Week 24	14.8 ± 2.1	16.3 ± 3.2	1.5 (−7.1–10.0)	.730
≥1 NYHA class improvement from baseline	Week 12	20/28 (71.4%)	16/22 (72.7%)	1.3% (−23.8%–26.4%)	.919
	Week 24	21/28 (75.0%)	14/15 (93.3%)	18.3% (−2.1%–38.7%)	.226

Values are n (%) or mean ± standard error for continuous variables.

CI, confidence interval; LVOTO, left ventricular outflow tract obstruction; NYHA, New York Heart Association

### Symptomatic response to mavacamten

**Figure 4:**
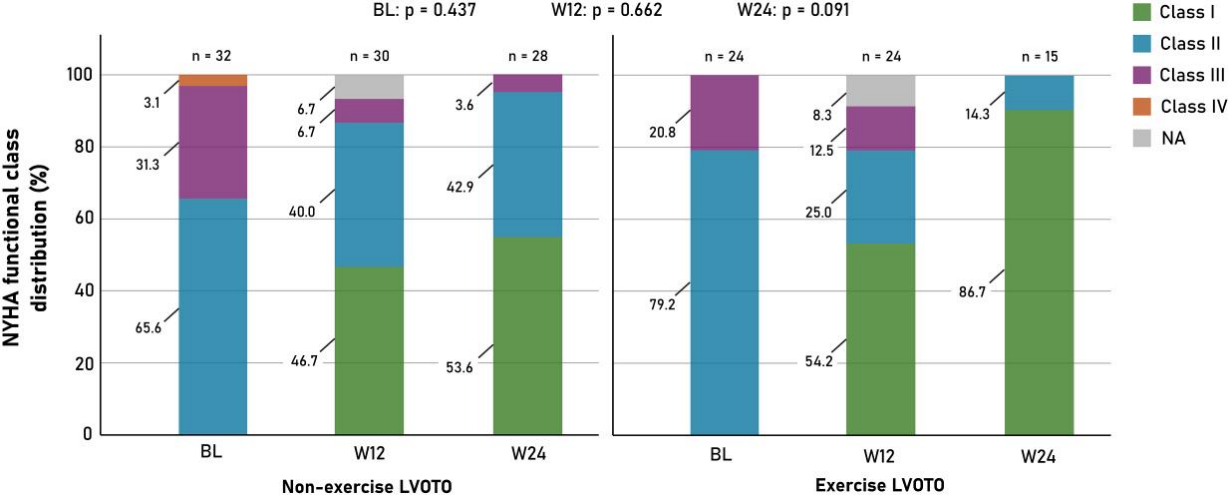
Longitudinal distribution of NYHA functional class during mavacamten therapy. Stacked bar charts illustrate the distribution of New York Heart Association (NYHA) functional classes at baseline (BL), 12 weeks (W12), and 24 weeks (W24) in patients who met treatment eligibility without exercise echocardiography (non-exercise LVOTO) and in those who qualified exclusively during exercise echocardiography (exercise LVOTO). Both groups demonstrate a progressive shift towards lower NYHA functional classes during follow-up, consistent with sustained symptomatic improvement. Numbers above the bars indicate the number of evaluable patients at each visit. *P*-values refer to between-group comparisons

NYHA functional class at 12 and 24 weeks was available in 50 of 54 patients (92.6%) and in all 43 patients (100%) with follow-up visits at those timepoints, respectively. By 12 weeks, improvement of at least one NYHA functional class was observed in 20 of 28 patients (71.4%) in the non-exercise LVOTO group and in 16 of 22 patients (72.7%) in the exercise LVOTO group (*P* = .919). By 24 weeks, symptomatic improvement was present in 21 of 28 patients (75.0%) in the non-exercise group and in 14 of 15 patients (93.3%) in the exercise group (*P* = .226). Longitudinal evaluation demonstrated a progressive shift towards lower NYHA classes across follow-up visits in the entire cohort (*P* < .001). There was no significant interaction between qualifying LVOTO group and follow-up visit (Visit*Group interaction, *P* = .161). As illustrated in *Figure 4*, there were no significant differences in NYHA functional class between groups at either follow-up timepoint.

### Mavacamten dose during follow-up

The distribution of mavacamten dose at 12 and 24 weeks is shown in *Table 4*. At 12 weeks, dose titration differed between groups (*P* = .012), with a higher proportion of patients in the non-exercise LVOTO group receiving doses of 5 mg or higher, whereas patients in the exercise LVOTO group were more frequently maintained on 2.5 mg. By 24 weeks, dose titration had progressed in both groups, and the distribution of mavacamten dose did not differ significantly between the non-exercise and exercise LVOTO groups (*P* = .146).

**Table 4:** Distribution of mavacamten dose at 12 and 24 weeks according to echocardiographic condition prompting treatment initiation

	Mavacamten dose	Non-exercise LVOTO	Exercise LVOTO	*P* value
Week 12	2.5 mg	8/30 (26.7)	17/24 (70.8)	.**012**
	5 mg	13/30 (43.3)	5/24 (20.8)	
	10 mg	8/30 (26.7)	2/24 (8.3)	
	15 mg	1/30 (3.3)	0/24 (0.0)	
Week 24	2.5 mg	6/28 (21.4%)	8/15 (53.3)	.146
	5 mg	12/28 (42.9)	2/15 (13.3)	
	10 mg	1/28 (3.6)	5/15 (33.3)	
	15 mg	1/28 (3.6)	0/15 (0.0)	

Values are n (%).

LVOTO, left ventricular outflow tract obstruction

## Discussion

In this retrospective, single-centre study of consecutive patients with oHCM treated with mavacamten, several important observations emerge: (1) a substantial proportion of patients required exercise echocardiography to demonstrate treatment-qualifying LVOT gradients for mavacamten; (2) patients with obstruction identified exclusively during exercise did not necessarily represent a milder clinical, functional, or physiological phenotype at baseline; and (3) mavacamten therapy resulted in comparable haemodynamic and symptomatic benefit irrespective of the presence of resting, Valsalva-provoked or exercise-induced only LVOTO.

Dynamic LVOTO is a hallmark of oHCM and may only become apparent under physiological provocation or exercise.^3,5,6,14^ In our cohort, more than 40% of patients met treatment eligibility criteria for mavacamten exclusively during exercise echocardiography. This underscores the critical importance of systematic exercise testing in symptomatic patients with suspected oHCM and borderline gradients at rest or during the Valsalva manoeuvre. These findings reinforce current guideline recommendations advocating to perform exercise echocardiography when resting gradients are insufficient to explain symptoms, and they highlight the risk of under-recognition of clinically relevant obstruction when exercise testing is omitted.^1,2,11^

A central finding of this study is that patients requiring exercise echocardiography to demonstrate LVOTO did not seem to exhibit a milder baseline disease phenotype. Although isolated exercise-induced LVOTO has been associated with a more favourable long-term prognosis,^7^ the present cohort indicates that, among symptomatic patients selected for mavacamten therapy, exercise-induced obstruction alone does not necessarily represent a less severe clinical phenotype. Despite having lower resting and Valsalva-provoked gradients by definition, these patients demonstrated symptom burden and functional impairment comparable to those with obstruction evident at rest or during Valsalva-provocation. Cardiopulmonary exercise supported this observation, revealing subnormal aerobic capacity in both groups, with peak VO_2_ values averaging approximately 70–80% of predicted, consistent with clinically meaningful exercise limitation and in line with prior CPET studies in symptomatic HCM.^15–19^ Ventilatory efficiency was similarly impaired, with Ve/VCO_2_ slopes exceeding normal thresholds. Stroke volume augmentation, assessed by VO_2_/HR, was likewise comparably reduced; this finding may be partially explained by blunted chronotropic responses in both groups, likely reflecting a combination of disease-related autonomic dysfunction and high background beta-blocker use in patients being assessed for mavacamten eligibility.^20,21^ Collectively, these CPET findings align with the clinical burden observed among patients meeting treatment eligibility regardless of the presence of resting, Valsalva-provoked or exercise-induced only LVOTO.

When examining exercise echocardiography, a striking observation was that peak exercise LVOT gradients were similar between groups despite markedly different resting and Valsalva-provoked gradients. This likely reflects, at least in part, routine clinical practice, in which patients with severe symptoms and overt resting obstruction are typically started on mavacamten without undergoing stress echocardiography, whereas those with less pronounced resting gradients undergo exercise testing as a part of further diagnostic workup. In our cohort, only 12 out of 32 patients (37.5%) in the non-exercise LVOTO group underwent exercise echocardiography, suggesting that the exercise-echo subset may represent a less symptomatic segment of the broader non-exercise LVOTO population. In addition, exercise-induced LVOTO is highly load-dependent and exhibits substantial intra-individual variability, with gradients influenced by preload, chronotropy, and contractility, which complicates direct comparisons between groups.

With respect to treatment response, mavacamten therapy was associated with marked improvement in LVOTO across the cohort. By 24 weeks, the vast majority of patients in both groups achieved non-obstructive LVOT gradients, and cross-sectional analyses adjusted for baseline demonstrated no significant between-group differences in residual Valsalva-provoked gradients at either follow-up timepoint. These results are consistent with the haemodynamic benefits reported in EXPLORER-HCM and VALOR-HCM,^8,10^ and extend those observations to patients with obstruction manifesting exclusively during exercise. Importantly, they indicate that the magnitude of haemodynamic improvement achieved with mavacamten, including attainment of non-obstructive LVOT gradients by 24 weeks, is comparable irrespective of whether treatment eligibility was established at rest, with Valsalva-provocation, or during exercise echocardiography. In the cross-sectional review of the individual timepoints, gradients in the exercise-induced only LVOTO group appeared to remain closer to baseline at week 12 and decreased more clearly by week 24. This early difference may, at least in part, reflect differences in dose titration during the initial treatment phase, as the first 12 weeks predominantly represent an up-titration period and patients in the exercise LVOTO group were more frequently maintained on lower mavacamten doses at 12 weeks, with similar dosing achieved by week 24 in both groups.

Symptomatic response mirrored the haemodynamic findings. Across the entire cohort, mavacamten therapy was associated with a significant improvement in NYHA functional class over follow-up. The proportion of patients achieving a clinically meaningful improvement of at least one NYHA class was substantial at both 12 and 24 weeks, with no significant differences between groups at either timepoint. Longitudinal analysis demonstrated a consistent trajectory of symptomatic improvement over time, without evidence of a different response between patients qualifying for treatment based on resting or Valsalva-provoked gradients and those requiring exercise echocardiography. Together, these findings indicate that mavacamten treatment resulted in durable symptomatic benefit both in the presence of resting, Valsalva-provoked or exercise-induced only LVOTO.

From a clinical perspective, these findings have important implications. Reliance on resting or Valsalva-provoked gradients alone may underestimate disease severity and delay access to disease-modifying therapy in a substantial subset of symptomatic patients. Our results challenge the notion that patients with exercise-induced only LVOTO represent a less severe or less treatment-responsive subgroup, and support the use of mavacamten in appropriately selected patients whose obstruction is unmasked exclusively during exercises. Moreover, they underscore the importance of comprehensive echocardiographic evaluation—including exercise echocardiography—not only for accurate disease characterization but also to ensure equitable access to disease-modifying therapy.

## Study limitations

Several limitations should be acknowledged. The retrospective, single-centre design limits generalizability, and the sample size, particularly at later follow-up timepoints, constrains statistical power for subgroup analyses. Additionally, treatment decisions and dose titration were guided by real-world clinical practice rather than a standardized protocol. Importantly, haemodynamic assessment at follow-up relied on resting and Valsalva-provoked gradients; follow-up exercise echocardiography was not performed. As dynamic LVOTO may persist during exertion despite normalization during the Valsalva manoeuvre, especially in patients whose obstruction was confined to exercise, the possibility of residual exertional gradients during therapy cannot be excluded. Nonetheless, the uniform application of treatment algorithms, reimbursement criteria, and systematic follow-up strengthen the internal validity of our findings.

## Conclusion

In conclusion, patients with oHCM who require exercise echocardiography to demonstrate LVOTO do not appear to exhibit a milder symptomatic or functional phenotype, and they derive haemodynamic and symptomatic benefit from mavacamten comparable to those with obstruction evident at rest or during Valsalva-provocation. These findings support comprehensive exercise-based assessment in oHCM and reinforce the role of mavacamten across the full spectrum of dynamic obstruction.

## Supplementary Material

xvag087_Supplementary_Data

## Declarations

### Data Availability

No data were generated or analysed for this manuscript.

### Pre-registered Clinical Trial Number

None supplied.
