# Supplementary material for: Role of provocation and exercise imaging for the identification of candidates for cardiac myosin inhibitors
Source: ESC Heart Fail. 2026 Mar 25;13(2):xvag087. doi: 10.1093/eschf/xvag087 (PMC13069892; doi:10.1093/eschf/xvag087)
Supplement: xvag087_Supplementary_Data [file xvag087_supplementary_data.docx]

**Supplemental Text 1**

Flowchart on the management of left ventricular outflow tract obstruction (adapted from the *2023 ESC Guidelines for the management of cardiomyopathies: Developed by the task force on the management of cardiomyopathies of the European Society of Cardiology (ESC)*^1^).

Disopyramide (Class I) OR **Mavacamten (Class IIa)**

Septal reduction therapies(Class I)

*Still symptomatic*

*Still symptomatic*

Verapamil (Class I) OR Diltiazem (Class I)

*Still symptomatic or intolerant/contra-indication to beta-blockers*

Beta-blockers (Class I)

Beta-blockers or verapamil may be considered (Class IIb)

No

Yes

Yes

Symptoms

Resting/provocable LVOTO ≥50 mmHg

**Supplemental Text 2**

In Belgium, reimbursement for mavacamten requires that treatment be initiated by a cardiologist working within a multidisciplinary heart failure or structural heart disease team. The medication must be dispensed through a hospital pharmacy.

Patients must meet all of the following clinical criteria:

1. Symptomatic obstructive hypertrophic cardiomyopathy with New York Heart Association (NYHA) class II or III symptoms.
2. Left ventricular wall thickness ≥15 mm, or ≥13 mm in the presence of a positive family history of hypertrophic cardiomyopathy (HCM).
3. Left ventricular ejection fraction ≥55% on echocardiography.
4. Left ventricular outflow tract (LVOT) obstruction documented by at least one measurement showing a peak LVOT gradient ≥50 mmHg at rest, after provocation (e.g. Valsalva), or during stress echocardiography; and a Valsalva-provoked LVOT gradient ≥30 mmHg at treatment initiation.
5. Oxygen saturation ≥90% at rest.
6. Prior treatment with a beta-blocker or non-dihydropyridine calcium-channel blocker (verapamil or diltiazem) is required and has to be continued, unless these agents were not tolerated.
7. Genotyping of CYP2C19 to determine metabolizer status is required before initiating therapy to guide dosing.
8. Pregnancy must be excluded in women of childbearing potential, and appropriate contraception is required.

Dosing recommendations follow the European label and depend on CYP2C19 metabolizer status: slow metabolizers initiate at 2.5 mg once daily, normal/intermediate metabolizers typically start at 5 mg once daily, with potential titration to 10 or 15 mg based on LVOT gradient and left ventricular function. Clinical and echocardiographic reassessment is performed at 4 and 8 weeks to guide dose adjustments. Treatment may be discontinued after 4–6 months if no clinical or hemodynamic improvement is observed.

**References**

1. Arbelo E, Protonotarios A, Gimeno JR, et al. 2023 ESC Guidelines for the management of cardiomyopathies: Developed by the task force on the management of cardiomyopathies of the European Society of Cardiology (ESC). *European Heart Journal*. 2023;44(37):3503-3626. doi:10.1093/eurheartj/ehad194
